# Cerebrospinal Fluid Genomic Profiling as an Adjunct in a Case of Intracranial Germinoma: Utility and Limitations

**DOI:** 10.3390/diagnostics16152394

**Published:** 2026-07-30

**Authors:** Edina Komlodi-Pasztor, Vindhya Udhane, Joseph Watson, Emily A. Sloan, Anousheh Sayah, Erini Makariou, Alexandra Larson, DeElegant Robinson, Kala F. Schilter, Qian Nie, Honey V. Reddi

**Affiliations:** 1 MedStar Georgetown University Hospital, Washington, DC 20006, USA; edina.komlodi-pasztor@medstar.net (E.K.-P.); joseph.watson@gunet.georgetown.edu (J.W.); emily.a.sloan@medstar.net (E.A.S.); anousheh.sayah@gunet.georgetown.edu (A.S.); makariev@gunet.georgetown.edu (E.M.); 2 Belay Diagnostics, Chicago, IL 60607, USA

**Keywords:** germinoma, intracranial germ cell tumors, CSF-NGS, 12p gain encompassing *KRAS*

## Abstract

**Background and Clinical Significance**: Primary central nervous system (CNS) germinomas are rare intracranial germ cell tumors that predominantly affect children and young adults. Histopathological evaluation remains the diagnostic gold standard; however, molecular characterization has increasingly contributed to tumor classification and biological understanding. Recent advances in cerebrospinal fluid tumor-derived nucleic acid (CSF-tNA) analysis provide an opportunity to identify tumor-associated genomic alterations through minimally invasive testing; however, its ability to distinguish CNS germ cell tumor subtypes has not been established and it should be interpreted as a complementary rather than standalone diagnostic tool. **Case Presentation**: We report a patient with an intracranial mass whose histopathological examination demonstrated features consistent with germinoma. Immunohistochemistry confirmed the diagnosis. Concurrent CSF-tNA analysis using Summit™ 2.0 assay revealed a 12p gain encompassing *KRAS*, along with copy number gains of chromosome arms 21q and 1q and losses of chromosome arms 11q, 13q, 5q, and 9q. Although these alterations overlap with molecular profiles reported in CNS germ cell tumors, including recurrent 12p gain and RAS/MAPK pathway activation, they cannot distinguish germinoma from other CNS germ cell tumor subtypes and should be interpreted in conjunction with histopathologic, radiographic, and clinical findings. **Conclusions**: This case illustrates the potential complementary role of CSF genomic profiling alongside conventional histopathology. CSF-based genomic analysis may contribute to supportive molecular information and may have future applications in disease monitoring rather than definitive tumor classification or subtype discrimination.

## 1. Introduction

Primary central nervous system (CNS) germ cell tumors (GCTs) are rare and biologically heterogeneous neoplasms, representing approximately 1–5% of primary intracranial tumors in children and adolescents. These tumors usually occur during the second decade of life and are more common in males [1,2,3,4]. Histopathologically, CNS GCTs are broadly classified into germinomas and non-germinomatous germ cell tumors (NGGCTs), the latter comprising yolk sac tumors, choriocarcinomas, embryonal carcinomas, teratomas and tumors with mixed histologies [5,6,7]. Distinction in subtype is clinically important, as it determines prognosis and treatment approaches [8,9].

Germinomas are the most common histologic subtype of CNS germinomas and are associated with a favorable prognosis [10,11]. The diagnosis of CNS germinoma is based on a combination of criteria including clinical presentation, neuroimaging, tumor biomarker evaluation, and histopathology. Pure germinomas characteristically have normal alpha-fetoprotein (AFP) levels and may show normal or mildly elevated beta-human chorionic gonadotropin (β-hCG) on serologic or CSF evaluation [12,13]. Elevation of AFP above 10 ng/mL or of β-hCG above 50–100 IU/L typically reflects a non-germinomatous component such as yolk sac tumor or choriocarcinoma. In the setting of characteristic imaging, such elevations may lead to a diagnosis without tissue biopsy, as outlined in international consensus guidelines [14]. Threshold values nonetheless differ between cooperative groups [10,15] and tumor markers can also rise in pure germinomas with syncytiotrophoblastic giant cells and in immature teratomas [5]. Additionally, definitive tissue-based diagnosis of CNS germinoma can be established through stereotactic or endoscopic biopsy [7,13].

Despite advances in neuroimaging and neuropathology, diagnosis of CNS germ cell tumors may still be delayed because their presenting symptoms frequently overlap with more common endocrine, ophthalmologic, or inflammatory disorders [16,17]. In published case series, median intervals from symptom onset to diagnosis are roughly 6 to 24 months, with individual delays of 5 years or more from when the first manifestation is isolated [18,19].

Currently, molecular profiling to inform diagnosis of germ cell tumors is not widely used. However, cerebrospinal fluid (CSF)-based liquid biopsy approaches using next-generation sequencing (NGS) analysis, an emerging, minimally invasive test, may be a useful adjunct tool in patients with suspected CNS germinoma, particularly when surgical biopsy is considered to be high risk or inconclusive [20]. Through analysis of circulating tumor-derived DNA (ctDNA) in CSF, NGS can detect molecular alterations that could support the diagnosis of germinomas [20]. CSF genomic profiling does not replace established diagnostic methods; rather, it enhances the understanding of tumor biology and supports future disease monitoring and therapeutic research, with its clinical value further informed by health economic outcomes research and incorporation into practice guidelines [21].

CNS germinomas commonly harbor activating somatic mutations in the receptor tyrosine kinase *KIT* gene, with recurrent hotspot alterations in exon 17 (notably substitutions at D816) that stabilize the activation loop and confer ligand-independent, gain-of-function signaling [22,23,24]. Large-scale genomic studies have shown that *KIT* mutations and alterations in RAS pathway genes (*KRAS*, *NRAS*, and related components) are typically mutually exclusive, supporting a model in which either upstream *KIT* activation or direct RAS pathway activation serves as the principal oncogenic driver in distinct molecular subsets of germinomas [23,25,26]. In addition to point mutations, focal amplification of *KIT* has also been reported [22,25]. Activating alterations in *AKT1*, *MTOR*, and other components of the PI3K pathway have also been identified and contribute to sustained pro-survival signaling and metabolic reprogramming [23,26]. At the chromosomal level, recurrent gain of chromosome 12p, an established hallmark of germ cell tumors, is detected in a subset of intracranial germinomas and likely increases the oncogenic drivers located on 12p, cooperating with *KIT/RAS* and *PI3K* pathway alterations to promote tumorigenesis [22,27]. Several molecular features of germinoma have drawn therapeutic interest, though none have displaced conventional chemoradiotherapy. Imatinib and dasatinib trials targeting recurrent *KIT* mutations have yielded no objective responses, largely because germinomas carry exon 17 *KIT* mutations that resist first-generation tyrosine kinase inhibitors (TKIs) [10].

Here, we present a case of primary CNS germinoma in which CSF genomic profiling identified chromosomal copy-number alterations, including 12p gain encompassing *KRAS*, in a tumor with histopathologic features diagnostic of germinoma. Alterations in *KRAS*, a component of the RAS/MAPK pathway, have been described in germinomas and contribute to tumor biology [22]. Detection of a 12p gain encompassing *KRAS* alteration in CSF illustrates the potential complementary role of CSF genomic profiling in this setting, while not independently establishing the diagnosis or distinguishing germinoma from other CNS germ cell tumor subtypes. A key limitation of this case is the absence of genomic profiling of the tumor tissue, which limits direct comparison between the biopsy specimen and the CSF-derived genomic findings. Therefore, the 12p gain encompassing *KRAS* and chromosomal copy-number alterations identified in the CSF cannot be definitively interpreted as representing the dominant tumor clone or intratumoral heterogeneity.

## 2. Case Presentation

The patient presented here is a 19-year-old female who was in her usual state of health when she began experiencing progressively worsening blurry vision and peripheral vision deficits. She reported a severe, pulsatile headache localized to the midline, rated 6/10 in intensity. The headache was exacerbated by standing and relieved by lying down. She described the pain as wave-like, without associated auditory symptoms, and noted pressure behind both eyes. During this episode, she experienced colorful visual phenomena with zigzag lines resembling a kaleidoscope, occurring without blurriness. The episode lasted several hours, accompanied by mild nausea but no vomiting. She took acetaminophen without relief, and the headache was resolved the following day.

Over the following weeks, her visual symptoms progressively worsened, including increasing blur and visual field deficits. She reported an episode of transient vision loss upon standing, though she retained consciousness. She presented to the emergency department at that time and received IV fluids, which temporarily improved symptoms. Over subsequent months, her visual field deficits continued to deteriorate. Additional history is notable for amenorrhea over the past two years, hair thinning, increased thirst, and anxiety. Laboratory testing showed elevated prolactin levels (45.6 ng/mL).

**Figure 1:**
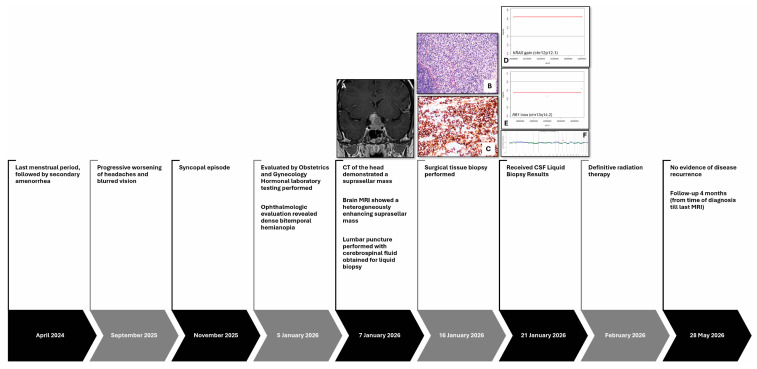
Clinical presentation and diagnostic evaluation timeline. (**A**) Post-contrast T1-weighted coronal magnetic resonance imaging (MRI). (**B**) Hematoxylin and eosin (H&E) stain showing sheets of epithelioid tumor cells with clear cytoplasm and prominent nucleoli admixed with small lymphocytes, consistent with germinoma (200× magnification). (**C**) D2-40 immunohistochemical stain demonstrating tumor cell positivity (200× magnification). (**D**) Low-pass whole-genome sequencing (LP-WGS) of cerebrospinal fluid (CSF) tumor-derived nucleic acid (tNA) demonstrated a chromosome 12p copy-number gain encompassing the *KRAS* locus. The ichorDNA plot showed a log2 ratio of 0.4412 for the affected region (*KRAS*). (**E**) LP-WGS demonstrating chromosome 13q copy-number loss encompassing the *RB1* locus. The ichorDNA plot showed a log2 ratio of −0.1269 for the affected region (*RB1*). Copy-number gains were defined using an analytical log2 ratio threshold of >0.2. (**F**) Genome-wide aneuploidy plot demonstrating chromosomal copy-number alterations identified by LP-WGS.

Three months after the initial presentation, optometry evaluation revealed bitemporal hemianopia. She was referred to a neurosurgeon for further assessment and subsequently sent to the hospital for urgent imaging. She presented to MedStar Georgetown University Hospital emergency department in January 2026 (Figure 1, Table 1). Imaging revealed a suprasellar mass on CT, and MRI demonstrated a heterogeneously enhancing sellar/suprasellar mass displacing the optic chiasm superiorly and partially encasing the left cavernous internal carotid artery (Figure 1A). Spine MRI showed no evidence of disease. Blood test revealed elevated β-hCG (52.1 IU/L (range 1.5–4.2 units) with normal AFP, and findings consistent with panhypopituitarism, as well as arginine vasopressin (AVP) deficiency. Endocrinology recommended pituitary hormone replacement.

A lumbar puncture was performed for CSF analysis and genomic profiling (Table 1). CSF was clear and colorless without xanthochromia; analysis showed RBC 46, total nucleated cells 29 (98% lymphocytes, 2% monocytes), glucose 43 mg/dL, and protein 61 mg/dL. Tumor markers were notable for markedly elevated β-hCG (239 IU/L; reference 0–3) with normal AFP (<1 ng/mL; reference 0–1). The patient was evaluated by endocrinology, neuro-oncology, pediatric oncology, and neurosurgery. She subsequently underwent transsphenoidal biopsy with partial debulking of the pituitary mass and tolerated the procedure well. Histopathologic examination showed sheets of epithelioid cells with prominent nucleoli and cytoplasmic clearing, and admixed small lymphocytes, diagnostic of germinoma (Figure 1B). There was no morphologic or immunohistochemical evidence of an NGGCT in the biopsy specimen. Immunohistochemical evaluation supported the histomorphologic impression of germinoma, with large tumor cells demonstrating diffuse positivity for CD117 and D2-40 (Figure 1C), focal positivity for PLAP, β-hCG, and TdT, and negativity for AFP and Glypican-3. SALL4 immunohistochemistry was not performed. There was no evidence of a mixed germ cell tumor, and genomic profiling of the CSF showed overlap with genomic features described in CNS germinoma and provided supportive, but not specific, molecular information consistent with the overall diagnostic context [13,22].

Genomic profiling of the CSF was performed in a CLIA-certified laboratory (Belay Diagnostics, Chicago, IL, USA) using the Belay Summit™ 2.0 test, a clinically validated comprehensive genomic profiling (CGP) test with a demonstrated clinical sensitivity of 96% and specificity of 98% [28].

The Belay Summit 2.0 CGP test evaluates single-nucleotide variants (SNVs), multi-nucleotide variants (MNVs), insertions/deletions (indels), copy number variants (CNVs), fusions, tumor mutational burden (TMB), and microsatellite instability (MSI) in 521 clinically relevant cancer-associated genes using NGS on CSF-derived nucleic acid [28]. Additionally, one low-pass whole-genome sequencing (LP-WGS) is used for robust detection of chromosome arm-level copy number (CN) variants (loss and/or gain) and focal gene alterations [29]. The cut-off for aneuploidy calls was abs(log2r) >0.09 based on the number of copies relative to the panel of normals [29]. For gene-level CNVs, a seg.mean (average number of copies of a specific DNA segment) cut-off value of ≥0.1 was set for gain (amplifications), and ≤0 to <0.1 was set for no CN change, with cut-off values for gene-level deletions being set at ≥−0.2 for loss (deletions) using clinically validated gene-specific quantitative polymerase chain reaction (qPCR) tests [29]. Results from the CSF liquid biopsy analysis demonstrated copy number gain of *KRAS* at the gene level. Evaluation of the *KRAS* locus using LP-WGS confirmed a chromosome 12p gain encompassing the *KRAS* locus (Figure 1D). Additionally, LP-WGS also showed loss of 13q encompassing the *RB1* locus (Figure 1E) along with a high degree of chromosomal instability, characterized by multiple chromosomal gains and losses. Specifically, gains of chromosomes 12p, 21q, and 1q and losses of chromosomes 11q, 13q, 5q, and 9q were observed (Figure 1F). This pattern of chromosomal copy-number alterations is more frequently observed in NGGCTs; however, similar but generally less extensive aneuploid changes can also be seen in germinomas [22,30].

The patient subsequently underwent definitive radiation therapy, which she tolerated well. Her treatment course was complicated by persistent panhypopituitarism. Nonetheless, post-radiation MRI demonstrated a significant interval reduction in the size of the sellar mass, with the residual pituitary gland measuring 0.3 cm and only minimal residual thickening of the pituitary stalk/hypothalamus (Table 1). The optic chiasm was no longer displaced, and the cavernous sinuses demonstrated a normal appearance. Follow-up spine MRI remained normal, with no evidence of metastatic disease (Figure 1).

## 3. Discussion

This case describes a 19-year-old female with progressive visual disturbance and panhypopituitarism who was ultimately diagnosed with a CNS germinoma. Her presentation highlights several characteristic but often underrecognized features of CNS germ cell tumors, particularly those arising in the sellar/suprasellar region in adolescents and young adults. Beyond these clinical features, geographic differences in CNS germinoma incidence have been documented, with higher rates reported in Asian populations than in Western populations, supporting the idea that host genetic, environmental, or developmental factors may contribute to susceptibility [3,31,32].

Suprasellar germinomas often cause endocrine dysfunction from hypothalamic–pituitary involvement, with symptoms like diabetes insipidus, growth hormone deficiency, hypogonadism, and hypopituitarism frequently appearing before imaging findings [33,34]. In this case, the patient’s two-year history of amenorrhea, hair thinning, and progressive excessive thirst and fatigue strongly suggests longstanding hypothalamic–pituitary dysfunction, particularly characteristic of suprasellar germinomas.

Compression of the optic chiasm explains the patient’s progressive visual field deficits. An important finding in this case was the elevated CSF β-hCG level (239 IU/L) and the biopsy findings, which demonstrated a pure germinoma without morphologic or immunohistochemical evidence of a NGGCT. While mild β-hCG elevation is well recognized in germinomas containing syncytiotrophoblastic giant cells, markedly elevated β-hCG levels may raise concern for a mixed germ cell tumor or an occult choriocarcinomatous component [35]. However, the immunophenotype strongly supported germinoma, with the large tumor cells demonstrating diffuse CD117 and D2-40 positivity, focal PLAP, β-hCG, and TdT expression, and negative AFP and Glypican-3 staining, without morphologic evidence of yolk sac tumor or other non-germinomatous elements [36]. SALL4 immunohistochemistry was not performed, and SALL4 could provide further confirmation of germ cell lineage, although it would not distinguish germinoma from NGGCT [37].

Ultimately, histopathologic evaluation following transsphenoidal biopsy confirmed germinoma. The decision to pursue transsphenoidal biopsy with partial debulking, rather than extensive resection, reflects the standard management paradigm for suspected germinomas, in which tissue confirmation is sufficient given the exquisite chemoradiosensitivity of these tumors. Aggressive surgical resection offers no survival benefit and carries a significant risk of additional endocrine, visual, and neurologic morbidity [10,38].

CSF-based genomic profiling identified a copy-number gain involving *KRAS*, confirmed by low-pass whole-genome sequencing as a chromosome 12p gain encompassing the *KRAS* locus. Recent studies have shown that three-dimensional genome organization and enhancer–promoter interactions regulate oncogene activation, suggesting that copy-number alterations may have functional effects beyond simple gene changes. In this context, the biological significance of the copy-number gain involving *KRAS* remains uncertain, and whether it influences RAS/MAPK pathway activity through broader regulatory mechanisms requires further investigation [39].

In addition, 13q loss encompassing *RB1* was identified, a recurrent genomic alteration that may affect components of the Rb/E2F regulatory pathway [40,41]. The specimen demonstrated a high degree of chromosomal instability, with gain of 12p, in particular, being a well-recognized cytogenetic hallmark of germ cell tumors, most commonly described in NGGCTs, although it may also be observed in germinomas [13,22]. Furthermore, 12p gain, complex chromosomal copy-number alterations, and an elevated CSF β-hCG level raise concern for NGGCT or mixed germ cell tumor biology. However, the biopsy demonstrated histomorphologic and immunohistochemical features consistent with germinoma [13]. This discordance may reflect sampling limitations of a small biopsy, or it may indicate overlap in genomic alterations between germinomas and NGGCTs. Although the current management of germinoma does not incorporate molecularly targeted agents, the recurrent involvement of the KIT/RAS/MAPK pathway has prompted early-phase clinical investigation of pathway-directed therapy, most notably inhibition of *KIT* with agents such as imatinib and dasatinib in newly diagnosed or recurrent disease [10]. Downstream MEK inhibition and combination targeted strategies remain a biologically plausible approach for refractory or recurrent disease but have not yet been evaluated clinically in CNS germ cell tumors [13]. This patient responded well to definitive radiation therapy, with marked radiographic regression of the sellar mass and resolution of optic chiasm displacement. Her treatment course was complicated by persistent panhypopituitarism requiring ongoing hormone replacement, while follow-up spine MRI remained negative for metastatic disease.

These findings highlight the potential utility of CSF profiling as a complementary tool for detecting recurrent genomic alterations in CNS tumors; however, the identified alterations, including MAPK pathway activation, are not specific to germinoma and may be observed across a broad spectrum of CNS neoplasms. Instead, these alterations show overlap with molecular profiles described in CNS germ cell tumors, as reported in prior studies, reflecting shared pathway activation rather than subtype specificity [22,42]. Accordingly, CSF-based genomic profiling in this case was not used in isolation to distinguish germinoma from NGGCTs, but rather as supportive evidence in conjunction with histopathology, serum tumor markers, and clinical–radiologic correlation.

In summary, this case highlights the classic clinical triad of endocrine dysfunction, visual compromise, and a midline suprasellar mass in a young adult with germinoma. It further demonstrates the value of integrating imaging, tumor markers, histopathology, and CSF-based genomic profiling as a complementary minimally invasive adjunct in the evaluation and diagnostic algorithm of CNS germ cell tumors. As molecular characterization of CNS germ cell tumors continues to evolve, CSF-based approaches may contribute to additional supportive information in selected clinical contexts; however, their future role in diagnostic algorithms remains to be established. A key limitation in this case presentation is the absence of a paired tumor sample for genomic comparison to confirm the molecular findings from CSF, suggesting that the molecular findings from CSF-based testing should be interpreted as supportive rather than definitive evidence of tumor genotype, more so in the case of germinomas wherein the molecular profiles of specific subtypes are still under investigation.

## 4. Conclusions

This case underscores the diagnostic challenge of CNS germ cell tumors in young adults, particularly when presenting with subtle visual and endocrine symptoms. Multimodal evaluation including imaging, laboratory studies, and histopathology facilitates accurate diagnosis. CSF genomic profiling is an emerging, minimally invasive tool. It cannot distinguish tumor subtypes on its own, but it can provide supportive molecular evidence and help identify tumor-associated genomic alterations. Before it can be used routinely in clinical practice, it will require standardized assays and further prospective study. At present, early clinical suspicion, prompt pituitary MRI, and close coordination between endocrinology, ophthalmology, and neuro-oncology remain the most practical ways to shorten diagnostic delay and reduce long-term complications in these young patients.

## Figures and Tables

**Table 1:** Clinical course, treatment, and molecular findings.

Category	Variable	Findings
**Presentation**	Age/sex at birth	19 Y/Female
	Initial symptoms	Amenorrhea, headache, and blurred vision
**Diagnostic evaluation**	MRI findings	Sellar/suprasellar mass with heterogeneous enhancement, superior displacement of the optic chiasm, and partial encasement of the left cavernous internal carotid artery
	Serum AFP	Normal
	Serum β-hCG	Elevated: 52.1 IU/L (reference range 1.5–4.2)
**CSF evaluation**	CSF AFP	Normal: <1 ng/mL (reference 0–1)
	CSF β-hCG	Elevated: 239 IU/L (reference 0–3)
**Pathologic diagnosis**	Histopathology	Germinoma: sheets of epithelioid cells with prominent nucleoli, cytoplasmic clearing, and admixed small lymphocytes
	Immunohistochemistry	Positive: CD117, D2-40 (diffuse); PLAP, β-hCG, TdT (focal); negative: AFP and Glypican-3
**CSF genomic profiling**	Specimen type	Cerebrospinal fluid
	Commercial assay	Summit™ 2.0
	CSF volume submitted (mL)	1
	DNA input (ng)	5–40
	Mean target coverage	>350×
**Genomic findings**	Copy-number profile	Extensive chromosome copy-number alterations consistent with chromosomal instability
	*KRAS* alteration	Chromosome 12p gain encompassing the *KRAS* locus
	*RB1* alteration	Chromosome 13q loss encompassing the *RB1* locus
	Final diagnosis	Primary CNS germinoma
**Treatment**	Chemotherapy	Not administered
	Radiotherapy	Definitive radiation therapy (4493.4 cGy, 25 fractions)
	Treatment complications	Persistent panhypopituitarism requiring endocrine management
**Follow-up** (**4 months from time of diagnosis until last MRI)**	Radiographic response	Significant reduction in sellar mass after radiation therapy
	Spine MRI follow-up	No evidence of metastatic disease
	Best radiographic response	Complete response
	Last follow-up	Last MRI 28/05/2026, no evidence of disease recurrence

## Data Availability

The original contributions presented in this study are included in the article. Further inquiries can be directed to the corresponding author.
